# Impact of a Fermented High-Fiber Rye Diet on *Helicobacter pylori* and Cardio-Metabolic Risk Factors: A Randomized Controlled Trial Among *Helicobacter pylori*-Positive Chinese Adults

**DOI:** 10.3389/fnut.2020.608623

**Published:** 2021-01-15

**Authors:** Kun Xue, Yuwei Liu, Kia Nøhr Iversen, Mohsen Mazidi, Zheng Qu, Chenglin Dong, Tayi Jin, Göran Hallmans, Per Åman, Anders Johansson, Gengsheng He, Rikard Landberg

**Affiliations:** ^1^Key Laboratory of Public Health Safety, Ministry of Education, Department of Nutrition and Food Hygiene, School of Public Health, Fudan University, Shanghai, China; ^2^Department of Biology and Biological Engineering, Chalmers University of Technology, Gothenburg, Sweden; ^3^Department of Gastroenterology, Shanghai Zhongye Hospital, Shanghai, China; ^4^Department of Clinical Laboratory, Shanghai Zhongye Hospital, Shanghai, China; ^5^Department of Public Health and Clinical Medicine, Umeå University, Umeå, Sweden; ^6^Department of Molecular Sciences, Swedish University of Agricultural Sciences, Uppsala, Sweden; ^7^Department of Odontology, Section of Cariology, Umeå University, Umeå, Sweden

**Keywords:** wholegrain, rye, cardiovascular disease, *Helicobacter pylori*, inflammation, LDL cholesterol, C-reactive protein, cereal fiber

## Abstract

**Background:** High dietary fiber intake has been associated with reduced risk of *Helicobacter pylori* infection and co-morbidities such as gastric cancer but also with reduced risk of cardiovascular disease. It has been suggested that fermented rye could affect *Helicobacter pylori* bacterial load and that high- fiber rye may be superior to wheat for improvement of several cardiometabolic risk factors, but few long-term interventions with high fiber rye foods have been conducted.

**Objective:** To examine the effect of high-fiber wholegrain rye foods with added fermented rye bran vs. refined wheat on *Helicobacter pylori* infection and cardiometabolic risk markers in a Chinese population with a low habitual consumption of high fiber cereal foods.

**Design:** A parallel dietary intervention was set up and 182 normal- or overweight men and women were randomized to consume wholegrain rye products containing fermented rye bran (FRB) or refined wheat (RW) for 12 weeks. Anthropometric measurements, fasting blood sample collection and ^13^C-urea breath test (^13^C-UBT) were performed at baseline and after 6 and 12 weeks of intervention as well as 12 weeks after the end of the intervention.

**Results:** No difference between diets on *Helicobacter pylori* bacterial load measured by ^13^C-UBT breath test or in virulence factors of *Helicobacter pylori* in blood samples were found. Low density lipoprotein cholesterol (LDL-C) and high sensitivity C-reactive protein (hs-CRP) were significantly lower in the FRB group, compared to the RW group after 12 weeks of intervention. The intervention diets did not affect markers of glucose metabolism or insulin sensitivity.

**Conclusions:** While the results of the present study did not support any effect of FRB on *Helicobacter pylori* bacterial load, beneficial effects on LDL-C and hs-CRP were clearly shown. This suggest that consumption of high fiber rye foods instead of refined wheat could be one strategy for primary prevention of cardiovascular disease.

**Clinical Trial Registration:** The trial was registered at www.clinicaltrials.gov, Identifier: NCT03103386.

## Introduction

*Helicobacter pylori* (*H. pylori*) is a common pathogen infecting approximately half the world's population (1). In China, the prevalence has been estimated to 56% but may differ substantially between regions (1). Epidemiological and clinical studies show that *H. pylori* infections lead to increased risk for several diseases including superficial gastritis, gastric and duodenal ulcers and gastric cancer (2–5). A growing number of experiments including *in vitro* and animal studies, as well as clinical trials have revealed the definite role of *H. pylori* in gastric carcinogenesis (6). *H. pylori* has been identified as a Group I carcinogen by the International Agency for Research on Cancer and is currently recognized as a major cause of gastric adenocarcinoma (7, 8). It has been estimated that about 78% of all gastric cancers can be attributable to *H. pylori* infection (9). Studies proposed that dietary patterns may play a crucial role in defining the final outcome of a *H. pylori* infection and diet may play an interactive role with *H. pylori* for development of gastric cancer (6, 10, 11). Probiotics, in the form of yogurts and supplements has shown a modest potential for *H. pylori* eradication (12). However, further clinical trials are required for improved understanding of the role of specific dietary components in *H. pylori* pathogenesis and development (13). Direct methods for investigating the development and progression of diseases in the upper GI-tract are typically invasive and are therefore difficult to use in a wide context (14, 15). Urea breath test (UBT) is a commonly used, non-invasive diagnostic tool to measure presence or absence of *H. pylori* infection. It measures the urease activity of *H. pylori* in the stomach after intake of labeled urea and detects ^13^C labeled CO_2_. It is a widely used diagnostic tool due to its high accuracy and low impact on patients, as compared to invasive methods such as endoscopic examinations of the gastrointestinal tract (14, 15).

Cardiovascular disease (CVD) accounts for 30% of global mortality and is the main cause of death overall with 17.3 million people dying from the disease every year (16). In China, CVD accounts for 45 and 43% of all deaths in rural and urban areas, respectively (17). Moreover, the prevalence of type 2 diabetes (T2D) is increasing rapidly all over the world. In China, almost 10% of all adults have T2D (18). Results from large cohort studies have clearly shown that healthy eating and an active lifestyle are important aspects for prevention and control of cardiometabolic diseases such as CVD and T2D (19–22). Based on observational data, high wholegrains and cereal fiber intakes have consistently been associated with lower incidence and mortality from non-communicable diseases, such as CVD and T2D (23–26). In addition to other well-established mechanisms (27), wholegrains and/or cereal fiber may have a favorable effect on local and systemic inflammatory processes (28–31). Reduced inflammation has been associated with lowered incidence and less adverse outcome in CVD and T2D, and biomarkers such as high sensitivity C-reactive protein (hs-CRP) and interleukin 6 have been forwarded as independent risk markers of CVD (32–36). Due to its higher fiber content, rye has been suggested to be superior to other cereals in terms of improving metabolic risk markers (37) with some supportive evidence from a few conducted studies (38–40).

Based on positive findings from *in vitro* studies showing reduced adherence and colonization of *H. pylori* in response to supernatants from fermented rye bran and feasibility of consuming fermented rye bran from a small pilot study in humans (www.epo.org, patent no.: EP1450626), the aim of the present study was to examine the effects of high-fiber wholegrain rye foods with added fermented rye bran vs. refined wheat as a strategy to reduce the *H. pylori* bacterial load among *H. pylori* positive men and women from a Chinese population, in a 12 week randomized controlled trial. We further investigated the effect of the intervention on cardio-metabolic risk factors, as secondary outcomes. We hypothesized that consumption of wholegrain rye with fermented rye bran, compared with refined wheat, would reduce *H. pylori* bacterial load and have a favorable effect on cardio-metabolic risk factors.

## Methods

A 12-week randomized parallel intervention study was conducted to compare the effects of wholegrain rye products with added fermented rye bran (FRB) vs. refined wheat (RW) products (control) on normal weight and overweight participants with *H. pylori* infection. Participants who completed the study were invited to participate in a follow up visit 12 weeks after completing the intervention. The study was conducted at Zhongye Hospital, Shanghai, in April-October 2015. Participants gave written consent before each visit to the clinic, as well as oral consent before each procedure. The study was approved by the Institutional Review Board at Fudan University and the study was conducted in accordance with the Declaration of Helsinki.

### Intervention Diets

The intervention diets consisted of four pieces of crisp bread and two portion packs of extruded cereal puffs per day, providing ~515 kcal/d (Table 1). Participants were free to consume the products at any time of the day to facilitate compliance. Products were packed in neutral packaging materials and participants were not informed which diet they were allocated to, but as wheat and rye products differ visually some participants might have guessed their allocation. Hospital staff conducting the outcome assessment were not aware of the participants' treatment allocation.

**Table 1:** Daily nutritional composition of fermented rye bran and refined wheat diets.

**Product**	**Amount (g)**	**Energy (kcal)**	**Protein (g)**	**Fat (g)**	**Carbohydrate^a^ (g)**	**Total fiber^b^ (g)**	**Soluble fiber (g)**	**Water (g)**	**Ash (g)**
**Fermented rye bran (FRB)**									
Crisp bread	44.8	145.5	4.3	1.2	23.9	11.1	3.9	3.2	1.2
Puffs	110.0	372.6	11.4	3.3	61.2	26.3	3.5	5.0	2.8
**Sum**	**154.8**	**518.1**	**15.7**	**4.5**	**85.1**	**37.4**	**7.4**	**8.2**	**4.0**
**Refined wheat (RW)**									
Crisp Bread	58.8	210.5	6.8	1.0	41.2	3.6	0.9	4.5	1.1
Puffs	84.0	302.7	9.7	1.6	58.1	8.6	4.0	4.9	1.4
**Sum**	**142.8**	**513.2**	**16.4**	**2.6**	**99.3**	**12.2**	**4.9**	**9.4**	**2.3**

a *Calculated by difference*.

b *Fiber content as analyzed by the Uppsala method with inclusion of fructans. The bold rows are the summary rows*.

The FRB products were produced from dried fermented rye bran, incorporated into wholegrain rye crisp bread product and extruded wholegrain rye puffs (25% on weight basis). The RW products were refined wheat crisp bread and extruded refined wheat puffs. The fermented rye bran (provided by Lantmännen, Stockholm, Sweden) was prepared by mixing rye bran with autoclaved tap water in a proportion of 1:5 w/w. *Lactobacillus plantarum* (DSMZ 13890) was cultured at 37°C for 24 h on MRS-agar, and thereafter it was suspended in autoclaved tap water and added in the rye bran-water mixture to final concentration of 10^5^ bacteria/mL. The mixture was incubated at 37° under gentle agitation for 24 h in 4 different batches. The final concentration of *L. plantarum* in the fermented rye bran was estimated by count of colony forming units to about 10^9^/mL. The fermented rye bran was dried and used in the production of products.

The crisp bread with fermented rye bran contained wholegrain rye flour (7.5 kg), fermented rye bran (2.5 kg), water (13.5 kg) and salt (100 g). The dough was mixed, baked in the oven for 10 min (gradient temperature) and dried for 30 min at 80°C, resulted in a final water content of 7%. The RW crisp bread contained wheat flour (10 kg), dextrose (400 g) water (3.6 kg), salt (133 g) and yeast (1.92 kg, 8% solution). The dough was mixed, pre-warmed for 35 min, baked in the oven for 7.5 min (gradient temperature) and dried for 30 min at 80°C, resulted in a final water content of 7%.

Both FRB and RW puffs were extruded on a co-rotating twin screw extruder APV MPF 19/25 (Baker Perkins Group Ltd., Peterborough, UK), with a screw speed of 450 rpm at 103°C. The feed rate of flour mixture was 50 g/min and water was added at a rate of 2.5–6 ml/min. After extrusion the puffs were oven dried for 45 min at 100°C. The flour mixture RW puffs consisted of refined wheat flour (98.2% w/w), salt and ascorbic acid, whereas the flour mixture for FRB puffs contained whole grain rye flour (74.2% w/w), fermented rye bran (25% w/w) and salt.

### Composition of Intervention Products

Samples of crisp bread and puffs were freeze dried and milled with a cyclone sample mill (Retsch, Haan, Germany). Dietary fiber content was analyzed according to the Uppsala method (41). Fructan, and starch content were analyzed using a K-FRUC kit (42), and a K-TSTA kit (43), respectively (Megazyme, Bray, Ireland). Crude fat was determined according to the method described in the Official Journal of the European Communities (1984) and protein according to the Kjeldahl method (*N* × 6.25). Dry matter was determined by drying the samples at 105°C for 16 h according to the AACC method 44-15A.

### Participants, Screening, and Randomization

Males and females, aged 20–70 years old, were invited to attend a screening visit at Zhongye Hospital. Participants were screened for *H. pylori* infection with ^13^C-urea breath test (^13^C-UBT), and as per manufacturer's recommendation a delta-over-baseline (DOB) value >4%0 were considered positive for *H. pylori* infection. *H. pylori* negative participants, as well as participants who reported smoking, use of medications (except for mild hypertension medication), chronic disease such as diabetes, CVDs or cancer, active peptic ulcers, pregnancy or planning pregnancy within the duration of the study, allergies or food intolerances or travel plans within coming 4 months were excluded.

The aim was that 50% of participants recruited should have BMI ≤ 24.00 (normal weight) and 50% should have BMI > 24.00 (overweight), as *H. pylori* infection has been shown to be associated with BMI (44, 45). BMI of 24 kg/m^2^ was chosen as cut-off as it has been shown to be the optimal distinction between overweight and normal weight in an adult Chinese population (46).

Eligible participants were randomly assigned to either the FRB or RW diet (1:1) by the study investigators in Shanghai. The randomization sequence was generated in Microsoft Excel and stratified for weight status (overweight or normal weight) to ensure equal distribution of normal weight and overweight subjects in each group. Participants in the two weight groups were ranked according to their ^13^C-UBT value obtained at the screening visit and every other person were then allocated to FRB or RW, respectively. Two participants chose to withdraw from the study shortly after randomization, before baseline, and two additional participants were chosen from the pool of screened volunteers to increase the sample size. Those two participants belonged to the same weight group and had ^13^C-UBT values in the same range as the withdrawn participants and were therefore allocated based on the withdrawn participants place in the ranked lists (both were allocated to FRB). One of these participants were however excluded at a later stage due having consumed antibiotics during screening and therefore not fulfilling the inclusion criteria. Furthermore, two participants, one in each intervention group, were mistakenly provided wrong foods at baseline and were re-allocated to the diet group matching the provided food.

### Study Design, Procedures, and Sample Size

Participants attended an examination visit at the clinic at baseline, and after 6 and 12 weeks of intervention. All participants who completed the 12-week intervention were invited to attend a follow-up visit 12 weeks after finalizing the intervention (24 weeks after baseline). Participants were instructed to avoid food and beverages, with the exception of water, from 18:00 the day before each examination.

At the examination, participants underwent anthropometric measurements and bacterial load was estimated using ^13^C-UBT. Blood samples were drawn into a 7 ml EDTA tube and 7 ml serum tube, following a 5 min rest in a supine position. EDTA tubes were centrifuged immediately after sampling, whereas serum tubes were kept at room temperature for 30 min before centrifugation. Samples were centrifuged 10 min at 4°C under 2,000 g and aliquoted into serum, plasma, buffy coat and red blood cells. The lipids, glucose, insulin and hs-CRP concentrations were analyzed with the fresh blood by the clinical diagnostic center of Zhongye Hospital. The remaining sample material was stored at −80°C for later analysis.

The primary outcome of the study was difference in DOB between treatments. Based on previous pilot studies we hypothesized that a 30% difference in DOB between treatments after 12 weeks could have clinical relevance. In order to detect a two-side difference between treatments of 30% using a σ = 919 (based on results from a pilot study), α = 0.05, with a statistical power of 80%, 75 participants in each group would be required. A drop-out rate of 20% was assumed and therefore 90 subjects per treatment arm were recruited.

### Anthropometric Measurements

At the screening visit, height was measured on a wall mounted stadiometer (Stature meter 2 m, Dongguan Nancheng Chengbida Electronic Equipment Factory, China) with the participant not wearing shoes. Body weight and body fat [determined by bioelectrical impedance analysis (BIA)], were measured on an electric scale [OMRON V-body HBF-701, OMRON Healthcare (China) Co., Ltd., China] with participants wearing light clothing and no shoes at baseline, week 6, 12 and 24.

A subgroup of 54 participants were selected on a volunteer basis to undertake a dual energy x-ray absorptiometry (DEXA) scan at all three occasions, in addition to the BIA, in order to validate the accuracy of the BIA measurements. The DEXA scan was conducted with a Lunar Prodigy Dual Energy X-ray Absorptiometer (GE healthcare, Illinois, USA) at Shanghai University of Sports under the same fasting conditions as the examinations.

### Urea Breath Test

A ^13^C-UBT (*Helicobacter pylori* tester SN 2 6918, Shenzhen Zhongnuke Headway Biotechnology Co., Ltd., China) was used as a non-invasive diagnostic tool to measure the presence of *H. pylori* at screening as well as at examination visits. Participants breathed into a gas collection bag before and 30 min after consuming a tablet containing 75 mg ^13^C labeled urea along with 80–100 ml of water. The carbon dioxide abundance in the exhaled air was detected by the instrument and the difference between the two time points were calculated and expressed as DOB in %0. As per the manufacturer's recommendation, DOB values above 4%0 were set as the cut-off and interpreted as positive indicator of *H. pylori* infection.

### Biochemical Analysis

Plasma glucose, total cholesterol (TC), high density lipoprotein cholesterol (HDL-C), low density lipoprotein cholesterol (LDL-C), triglycerides and Apolipoprotein A/B/E/(a) were measured by a 7180 Clinical Analyzer (Hitachi, Japan). Serum hemoglobin A1c was measured by the VARIANT™ II TURBO Hemoglobin Testing System (Bio-Rad, USA). Serum insulin and C-peptide were measured by immunoassay using Cobas E601 analyzer (Roche, USA). Serum hs-CRP was measured with IMMAGE® 800 immunochemistry system (Beckman Coulter, USA). HOMA-IR was calculated as follows: fasting glucose (mmol/L) × [fasting insulin (pmol/L)/6]/22.5 (47). Plasma alkylresorcinols (AR) were analyzed by gas chromatography–mass spectrometry as a measure of wholegrain and bran intake from rye and wheat sources (48).

Antibodies directed against virulence factors were measured in serum, as a supportive measure of the *H. pylori* infection diagnosis made by ^13^C-UBT. Antibodies against the following virulence factors were measured: cytotoxin-associated gene A (anti-CagA), vacuolating cytotoxin A (anti-VacA), urease A (anti-UreA) and urease B (anti-UreB). Analysis was conducted by Western blot (Typing detection kit for antibody to *Helicobacter pylori*, Shenzhen blot biotech Co., Ltd., China) according to the instructions provided by the manufacturer.

### Statistical Analysis

All statistical analyses followed a per-protocol approach inherent to the design of the study. All outcomes, except serology, were analyzed using an unadjusted ANOVA model (Model 1) and an ANCOVA model (Model 2) adjusted for weight change (from baseline to week 6 and baseline to week 12, respectively), age and sex. Analyses of body weight and fat percentage were not adjusted for weight change. Models were visually inspected using quantile-quantile-plots and residual plots and response variables were log transformed if residuals were non-normally distributed. Estimates are presented as mean ± standard deviation. Median was used when estimates appeared skewed in a histogram. Stratified analysis dividing participants into overweight and overweight (status at baseline) was conducted by the same procedure as the unstratified analyses.

McNemar's test was used to evaluate the change in the number of seropositive participants from baseline to weeks 6, 12, and 24, respectively.

Analyses of hs-CRP was conducted after excluding observations ≥1 mg/L, as concentrations above this could indicate an acute infection (e.g., influenza, common cold, bacterial infections etc.) rather than low grade/chronic inflammation related to metabolic status/risk. Hs-CRP measures below the detection limit (<12.7% of the observations) was imputed the value of the detection limit (0.02 mg/L).

For the validation of BIA against DEXA pearson's correlation coefficient were calculated for each of the three occasions (baseline, weeks 6 and 12).

All data presented in tables are unadjusted data. *p*-values < 0.05 are considered statistically significant. Complete-case analysis was defined as the primary analysis strategy. The statistical analyses were carried out using R Studio (version 3.3.0 (2016-05-13), for Windows 7), and all estimates were derived using the plyr-package (version 1.8.3). Figures were made with GraphPad Prism 7 (version 7.04 (2017-11-28), for Windows 7).

## Results

**Figure 1:**
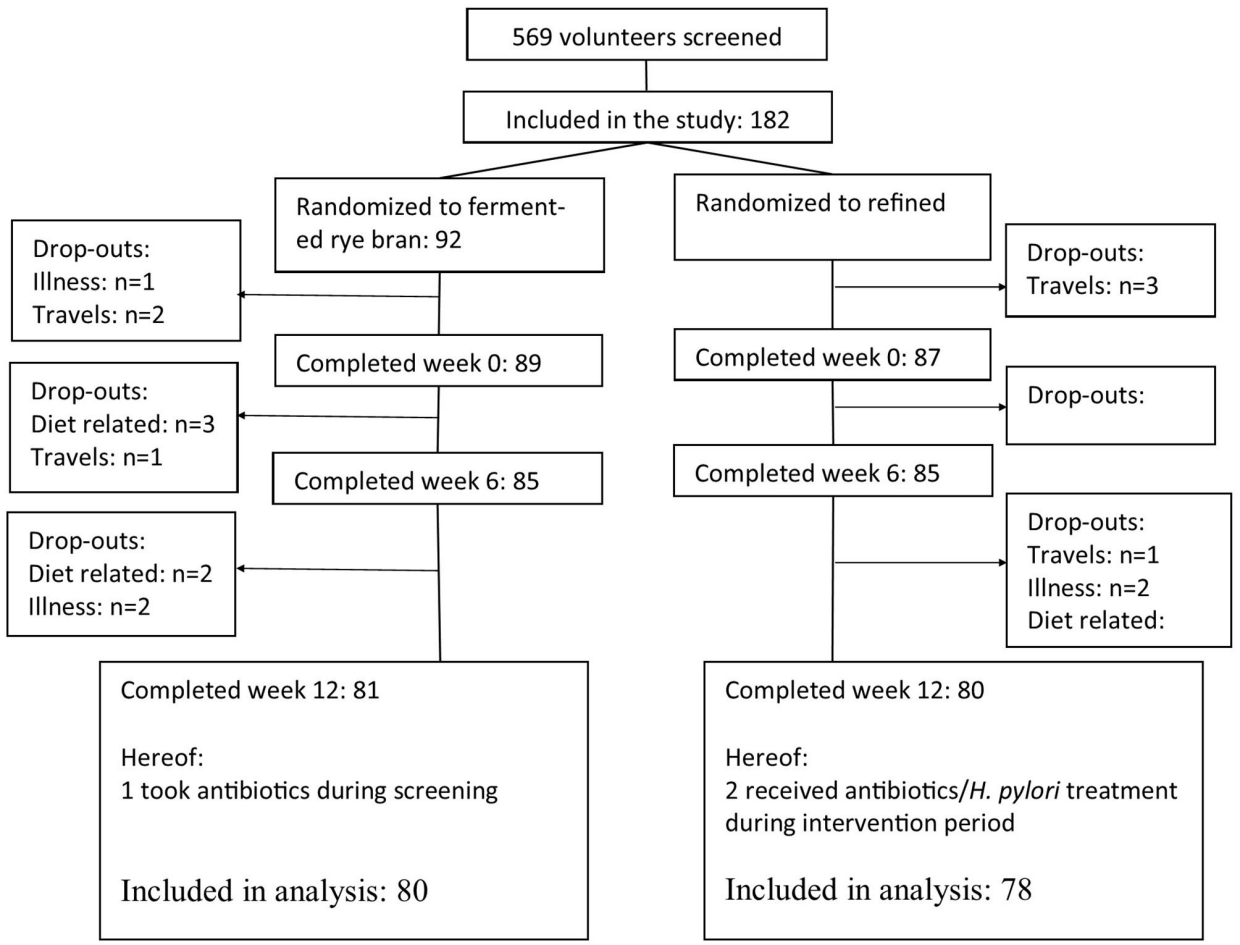
Flow sheet of participants.

In total, 569 men and women were screened by ^13^C-UBT to determine whether they were *H. pylori* positive or not (Figure 1). In total, 316 participants (55%) were found to be *H. pylori* positive (with DOB > 4%0). After exclusion of participants that did not fulfill additional inclusion/exclusion criteria, 182 *H. pylori* positive participants, hereof 94 normal weight and 88 overweight, were included in the study and randomized to either FRB (*n* = 92) or RW (*n* = 90).

Among 92 participants randomized in the FRB, 81 participants (88%) completed the 12-week intervention. The corresponding figure for the RW was 80 out of 90 randomized participants that completed the study (89%). The reasons for dropout were similar in both groups and included illness, travels, difficulties to adhere to the diet, use of antibiotics and treatment against *H. pylori* infection (Figure 1). Additionally, three participants (FRB:1, RW:2) were excluded from all data analysis as they reported use of antibiotics during screening and/or intervention period, which was not allowed according to the study protocol.

In total, 158 completed the study and were included in the analysis of the results (Table 2). Some participants were not fasted at the examinations (one in FRB at baseline, 3 in RW at week 6), and their data was therefore not included in the analysis of outcomes that could be sensitive to fasting status. In total, 125 returned for the follow-up visit at week 24, however one without having a blood sample taken. Additionally, 17 participants (FRB: 11, RW: 6) were not fasted at and their data was therefore not included in the reposted results.

**Table 2:** Baseline characteristics of the participants who completed the 12-week intervention.

	**Intervention groups**					
	**Fermented rye bran (FRB)**			**Refined wheat (RW)**		
	**All (*n* = 80)**	**Overweight^b^ (*n* = 41)**	**Normal weight^c^ (*n* = 39)**	**All (*n* = 78)**	**Overweight^b^ (*n* = 34)**	**Normal weight^c^ (*n* = 44)**
Age (years)*^a^*	44.5 ± 12.5	44.9 ± 11.8	44.1 ± 13.4	45.4 ± 14.0	48.9 ± 11.7	42.7 ± 15.1
BMI (kg/m^2^)*^a^*	24.3 ± 3.9	27.2 ± 3.1	21.4 ± 1.8	24.1 ± 4.2	27.9 ± 3.2	21.1 ± 1.8
Female/male (*n*)	62/18	28/13	34/5	64/14	27/7	37/7

a *Values expressed as mean and standard deviation*.

b *BMI > 24 kg/m^2^*.

c *BMI ≤ 24 kg/m^2^*.

Compliance, assessed by plasma AR concentrations (Table 3), indicated poor compliance in the FRB group and compliance apparently dropped after 6 weeks of intervention. The compliance in the RW group is difficult to determine using AR, since both the background diet and the refined intervention are expected to lead to low plasma AR concentrations.

**Table 3:** Plasma total alkylresorcinol concentration and the C17:0/C21:0 alkylresorcinol ratio.

	**Base-line**	**Week 6**	**Week 12**
**Total alkylresorcinol (nmol/L)^a^**			
FRB (*n* = 77/80/78)	2.8 (2.7)	28.0 (81.6)	16.1 (56.9)
RW (*n* = 79/74/79)	2.5 (3.7)	4.1 (4.7)	3.7 (4.4)
**C17:0/C21:0^a^**			
FRB (*n* = 13/72/62)^b^	0.10 (0.14)	0.67 (0.52)	0.56 (0.53)
RW (*n* = 19/30/17)^b^	0.05 (0.03)	0.11 (0.31)	0.08 (0.06)

a *Values are median (interquartile range)*.

b *C17:0 was below the detection limit in many participants, which is indicative of little or no rye intake*.

Body weight and body fat remained stable throughout the intervention for all participants, with no differences between groups (Table 4). The assessment of body fat percentage using BIA was validated against body fat percentage measured by DEXA in a subgroup of participants. The fat percentages measured by the two methods were well correlated (Pearson's correlation coefficient ≥ 0.84, *p* > 0.001, Supplementary Figure 1 in additional file 1), suggesting that use of BIA for body fat was an acceptable tool in this population.

**Table 4:** Effects on anthropometrics and clinical outcomes.

	**Week 0**	**Week 6**	**Week 12**	**Δ** **Between groups, week 6**		**Δ** **Between groups, week 12**	
				**Model 1**	**Model 2**	**Model 1**	**Model 2**
**Body weight (kg)**							
FRB	64.2 ± 13.3	64.4 ± 13.4	64.4 ± 13.6	0.610	0.587	0.696	0.635
RW	63.0 ± 14.3	62.6 ± 14.6	62.6 ± 14.4				
**Body fat (%)**							
FRB	30.8 ± 5.9	30.3 ± 6.2	30.0 ± 5.4	0.600	0.529	0.719	0.692
RW	31.5 ± 5.1	30.8 ± 5.9	30.5 ± 5.5				
**Triglycerides (mmol/L)**							
FRB	1.14 ± 0.55 (1.06)	1.25 ± 0.69 (1.08)	1.49 ± 1.08 (1.25)	0.685	0.821	0.207	0.162
RW	1.21 ± 0.61 (1.06)	1.33 ± 0.71 (1.11)	1.43 ± 0.85 (1.24)				
**Glucose (mmol/L)**							
FRB	5.50 ± 0.86	5.54 ± 0.57	5.40 ± 0.76	0.817	0.733	0.605	0.475
RW	5.74 ± 0.98	5.64 ± 0.83	5.58 ± 0.90				
**Insulin (pmol/L)**							
FRB	70.0 ± 38.2 (59.3)	73.2 ± 46.9 (59.7)	81.6 ± 55.7 (65.4)	0.220	0.200	0.621	0.830
RW	72.2 ± 39.9 (61.0)	74.9 ± 55.3 (57.3)	76.1 ± 40.7 (63.6)				
**C-peptide (nmol/L)**							
FRB	0.70 ± 0.25 (0.65)	0.72 ± 0.26 (0.68)	0.85 ± 0.38 (0.72)	0.420	0.431	0.336	0.516
RW	0.74 ± 0.28 (0.70)	0.76 ± 0.33 (0.65)	0.82 ± 0.28 (0.77)				
**HOMA-IR**							
FRB	2.9 ± 1.8 (2.4)	3.03 ± 1.99 (2.40)	3.4 ± 2.6 (2.6)	0.250	0.241	0.765	0.999
RW	3.1 ± 2.0 (2.6)	3.29 ± 2.92 (2.28)	3.2 ± 2.3 (2.8)				
**HbA1C (%)**							
FRB	5.50 ± 0.39	5.77 ± 0.39	5.78 ± 0.36	0.112	0.154	0.159	0.141
RW	5.57 ± 0.59	5.85 ± 0.53	5.87 ± 0.53				
**Apolipoprotein A (g/L)**							
FRB	1.38 ± 0.23	1.36 ± 0.22	1.35 ± 0.20	0.619	0.463	0.377	0.518
RW	1.36 ± 0.22	1.34 ± 0.23	1.36 ± 0.23				
**Apolipoprotein B (g/L)**							
FRB	1.13 ± 0.28	1.17 ± 0.28	1.15 ± 0.28	0.358	0.336	0.088	0.102
RW	1.13 ± 0.24	1.20 ± 0.20	1.22 ± 0.26				
**Apolipoprotein A/Apolipoprotein B**							
FRB	1.29 ± 0.36	1.25 ± 0.44	1.24 ± 0.37	0.175	0.109	0.244	0.203
RW	1.26 ± 0.31	1.15 ± 0.27	1.17 ± 0.36				
**Apolipoprotein E (g/L)**							
FRB	33.08 ± 5.75	33.18 ± 5.67	33.55 ± 5.69	0.514	0.465	0.485	0.347
RW	33.78 ± 5.43	33.13 ± 5.47	33.6 ± 5.27				
**Lipoprotein (a) (mg/L)**							
FRB	213.9 ± 182.3 (151.0)	240.8 ± 223.7 (142.0)	259.8 ± 272.9 (141.5)	0.457	0.371	0.643	0.499
RW	180.2 ± 171.7 (121.5)	208.0 ± 257.4 (113.5)	211.8 ± 235.3 (116.5)				
**Zonulin (pg/ml)**							
FRB	273.30 ± 88.37 (255.36)	358.10 ± 91.50 (353.77)	364.24 ± 94.17 (349.53)	0.954	0.847	0.639	0.727
RW	286.86 ± 97.00 (280.26)	356.29 ± 99.00 (329.16)	360.12 ± 101.63 (356.93)				

*Number of participants, when deviating from 80/78 (FRB/RW). Body weight: wk6 = 80/74, wk12 = 79/78, body fat %: wk0 = 78/77, wk12 = 79/78, insulin: wk12 = 79/77, C-peptide: wk12 = 79/76, HOMA-IR: wk12 = 79/77, lipoprotein (a): wk6 = 77/74, wk6 = 77/74, wk12 = 78/78, zonulin: wk6 = 78/75, wk12 = 79/78*.

*All data is mean ± SD (median)*.

*HOMA-IR, homeostasis model assessment insulin resistance; HbA1C, hemoglobin A1c*.

*n: FRB = 80, RW = 78 unless otherwise stated*.

Statistically significant differences are indicated by *p < 0.05.

### Bacterial Load- the Primary Outcome

**Figure 2:**
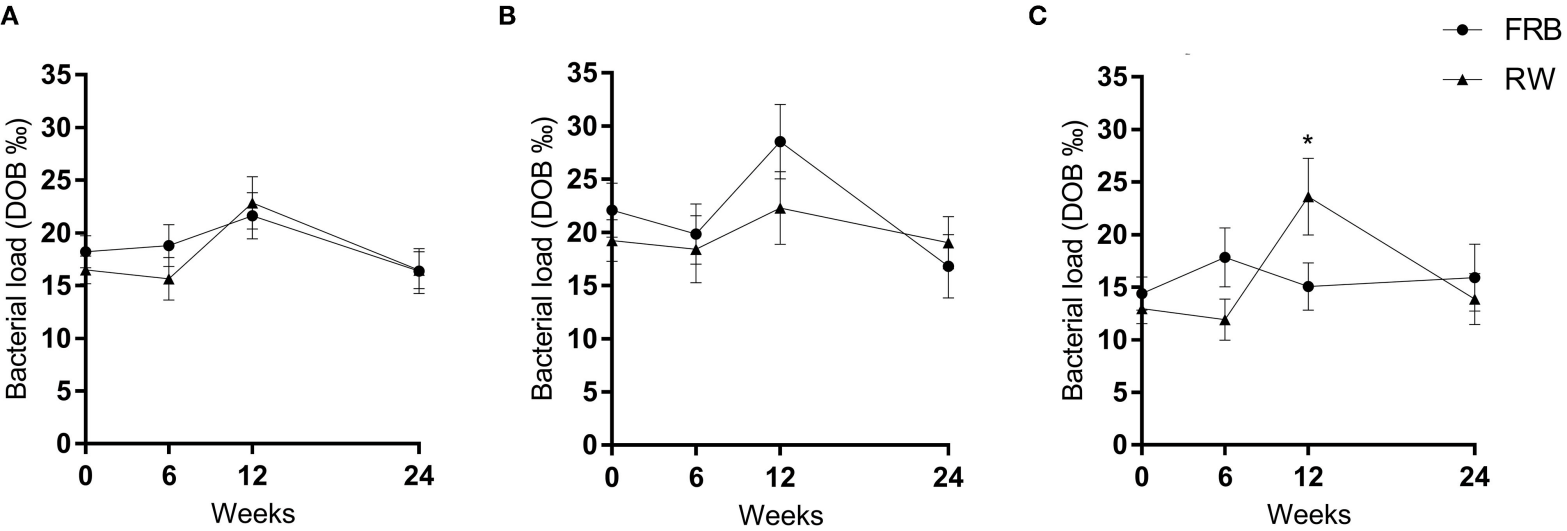
Bacterial load measured ^13^C-urea breath test at base-line, after 6 and 12 weeks of intervention and 12 weeks post intervention (week 24) in the fermented rye bran (FRB) and in the refined wheat (RW) group among all participants **(A)** and across normal weight participants **(B)** and overweight participants **(C)**. Data is mean ± SEM. DOB, delta-over-baseline. Statistically significant differences are indicated by *p < 0.05.

There was no overall difference of bacterial load assessed by ^13^C-UBT between groups when comparing data from completers after 12 weeks of intervention (Figure 2). When stratifying the analyses for overweight and normal weight participants, we found a higher bacterial load among overweight participants consuming RW, compared to FRB, after 12 weeks of intervention suggesting an adverse effect of RW (Figure 2).

Bacterial load at baseline differed between overweight and normal weight participants (mean DOB was 14.4%0 in overweight participants and 22.2%0 in normal weight participants, *p* = 0.01).

### Serology

Overall, there were no consistent pattern in changes in the proportion of participants who were measured positive for virulence factor during the intervention across the two treatment groups (Supplementary Table 1 in additional file 1). After 12 weeks of intervention anti-UreA decreased in participants consuming FRB (73 vs. 68%, *p* < 0.05) but remained stable in participants consuming RW. Anti-CagA increased in a subgroup on normal weight participants consuming RW (59 vs. 79%, *p* < 0.05) but remained stable across other subgroups. There was no change in the fraction of participants measured positive for anti-VacA and anti-UreB during the 12 week intervention. At follow up (week 24), the proportion of participants measured positive for anti-CagA (65 vs. 46%, *p* < 0.05), anti-VacA (54 vs. 39%, *p* < 0.05), anti-UreA (70 vs. 53%, *p* < 0.05) and anti-UreB (91 vs. 75%, *p* < 0.05) was reduced in the RW group, compared to baseline, whereas the number of positive subjects in the FRB group had only reduced for anti-UreA (87 vs. 65%, *p* < 0.05) (Supplementary Table 2 in additional file 1).

### Glucose Metabolism and Insulin Sensitivity

We found no statistically significant difference between interventions in any markers of glucose metabolism or insulin sensitivity (Table 4) for all or in any of the subgroups stratified for weight (Supplementary Tables 3, 4 in additional file 1).

### Blood Lipids

**Figure 3:**
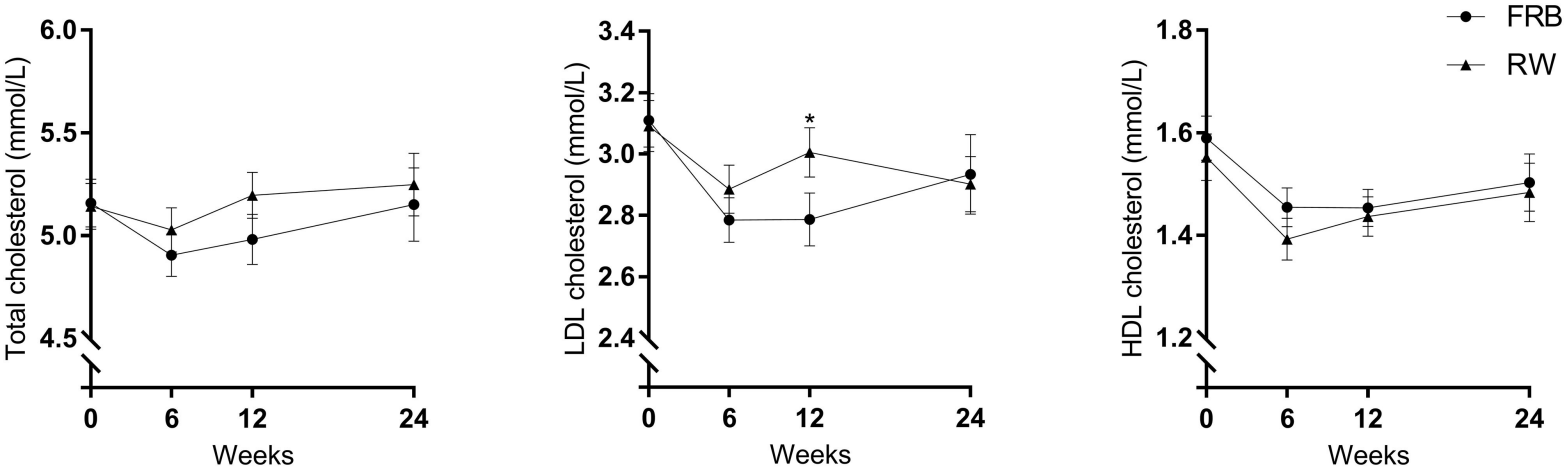
Plasma total cholesterol, low density lipoprotein (LDL) cholesterol and high density lipoprotein (HDL) cholesterol at base-line, after 6 and 12 weeks of intervention and 12 weeks post intervention (week 24) in the fermented rye bran (FRB) and in the refined wheat (RW) group among all completed cases. Data is mean ± SEM. Statistically significant differences are indicated by *p < 0.05.

No difference in TC or HDL-C was found between the two treatments or within groups, overall or for normal or overweighed participants separately, at base line (Figure 3). However, LDL-C was lower in the group consuming FRB, compared to the RW group after 12 weeks of intervention (2.79 ± 0.77 vs. 3.01 ± 0.71, *p* = 0.007 in model 1, *p* = 0.011 in model 2) (Figure 3). The effect had disappeared at the follow-up visit at week 24, when participants consumed their habitual diet (Supplementary Table 5 in additional file 1). Weight stratified analyses showed that the difference in LDL-C was mainly found among normal weight participants, but not among overweight participants, though baseline concentration were similar among the two weight groups (Supplementary Figures 3, 4 in additional file 1).

### Biomarkers of Inflammation and Leaky Gut

**Figure 4:**
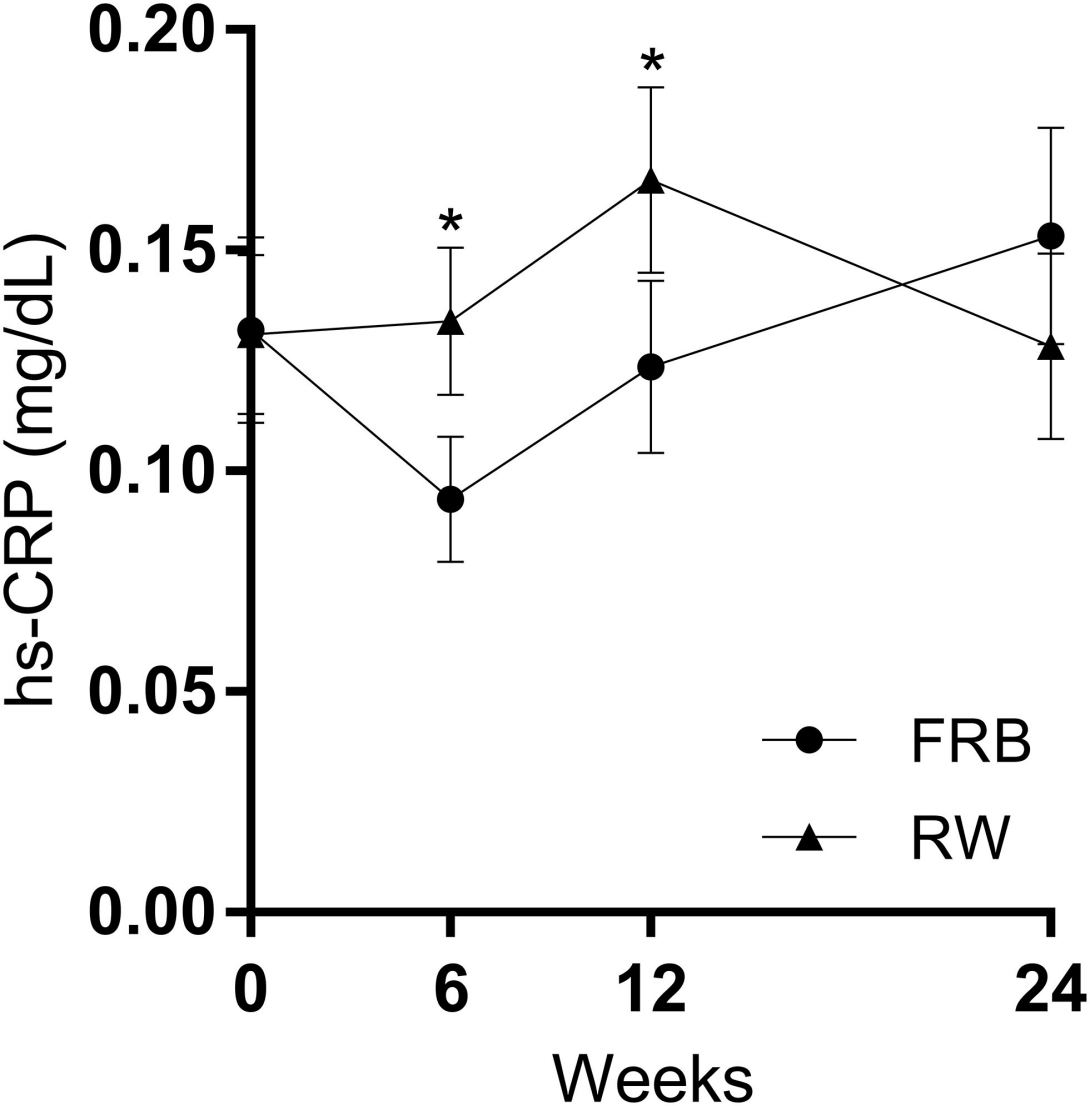
Serum high-sensitive C-reactive protein (hs-CRP) at base-line, after 6 and 12 weeks of intervention and 12 weeks post intervention (week 24) in the fermented rye bran (FRB) and in the refined wheat (RW) group among all completed cases. Observations ≥1 mg/dL were removed (*n* = 6 in FRB and *n* = 5 in RW, 1.9% of all observations). Data is mean ± SEM. Statistically significant differences are indicated by *p < 0.05.

Hs-CRP was significantly lower in participants consuming FRB instead of RW after 12 weeks of intervention (Figure 4). This effect was found among overweight, but not normal weight participants (Supplementary Tables 3, 4 in additional file 1). No differences in zonulin concentrations were found between groups, but the zonulin concentration increased in both groups during the intervention compared to base-line (Table 4). At the week 24 follow-up visit the zonulin concentration was significantly higher in the RW group, compared to FRB group, but the concentration had increased in both groups during the follow-up period (Supplementary Table 5 in additional file 1).

## Discussion

In the present study we investigated and found no effect of a 12-week dietary intervention with FRB vs. RW on *H. pylori* bacterial load, measured by ^13^C-UBT according to per-protocol analysis. This was supported with data showing no difference in measured virulence factors, which remained stable throughout the intervention and showed no difference between diets. In contrast, LDL-C and hs-CRP were lower after 12 weeks of FRB vs. RW consumption suggesting beneficial effects of high fiber rye consumption on important cardiometabolic risk markers.

### Effects on *H. pylori* Bacterial Load

The lack of an apparent effect on bacterial load indicated by ^13^C-UBT after dietary treatments is difficult to interpret but was supported by the overall no effects on *H. pylori* virulence factors, which altogether suggest no effect on *H. pylori* infection. The underlying hypothesis of using UBT was that reduction of bacterial load may have beneficial effects on *H. pylori* infection and that fermented rye bran could reduce the bacterial load through inhibition of adherence of *H. pylori* at the stomach mucosa. We assumed that ^13^C-UBT can be used to assess and monitor the severity of *H. pylori* infection in a longitudinal study setting rather than just determine whether a subject is positive or negative for *H. pylori*. The clinical use is primarily aimed toward determine whether a subject is positive or negative for *H. pylori*, rather than tracking the severity of the infection (49). However, some studies have shown cross-sectional associations between UBT results and severity of infection, assessed by endoscopic examinations and biopsies, which could indicate that UBT can be used to estimate the severity of *H. pylori* infection (50–52). On the contrary, other studies have not been able to show this association (53, 54), and it remains to be proven whether UBT can be used to detect changes in infections in longitudinal design such as the present study. The absence of the validation of UBT to track infection rate over time is an important weakness of the present study which may explain the lack of difference between the treatment groups. UBT test result has been shown to differ according to factors such as age and gender (55), as well as the use of antibiotics and proton pump inhibitors, gastric pH, bleeding in the gastrointestinal tract, gastric emptying rate and food consumption prior to the test, and the matrix in which the urea tablet is consumed (14, 15). We have attempted to control these factors by excluding participants that did not comply with fasting procedures, had gastric ulcers or reported use of drugs that could have affected the results. Indeed, the bacterial load was found to be higher in a subgroup of overweight participants consuming RW, compared to overweight participants consuming FRB, after 12 weeks of intervention, however there were no difference between groups when assessing the entire study population. Furthermore, the difference between interventions among overweight participants appears to be mainly due to an adverse effect of RW, rather than a positive effect of FRB, on bacterial load and therefore does not support an effect of the tested FRB products on *H. pylori* infection. Few dietary interventions targeting *H. pylori* infection have been conducted and it is therefore difficult evaluate whether 12 weeks is a sufficient time-frame to evaluate the potential of a dietary intervention to affect *H. pylori* infection. Valid evaluation of treatment effect at 4–6 weeks after pharmaceutical eradication of *H. pylori* infection have been shown for ^13^C-UBT readouts (49). Treatment with probiotics in time range of 4–6 has likewise been shown to reduce UBT readouts, despite probiotics only showing modest potential for eradication of *H. pylori* (12). However, it has not been established how fast *H. pylori* infection will respond to a dietary intervention such as the one in the current study.

Analysis of virulence factor antibodies is less sensitive to the factors mentioned above, however antibodies have been shown to persist more than 1 year after eradication of *H. pylori* and may not allow distinction between past and present infection in the relatively short timeframe of the present study and is not recommended for monitoring of *H. pylori* infection (15, 56, 57). Inclusion of endoscopic examinations, either alone or in combination with ^13^C-UBT, would likely have provided a stronger measure of the bacterial load and the severity of *H. pylori* infection. However, due to the invasive nature of endoscopic examination it was not possible in the context of this study.

Interestingly, ^13^C-UBT values at baseline were higher among normal weight participants, compared to overweight participants, which could indicate that the bacterial load was higher among normal weight participants. This is in line with the findings of Eisdorfer et al. who found an inverse association between DOB values and BMI in a cross sectional study of 76,000 *H. pylori* infected participants (55). Furthermore, increase in BMI following eradication therapy has been observed in some studies (58, 59), as well as an inverse association between the hunger inducing hormone ghrelin and *H. pylori* infection (60, 61). On the contrary, observational studies have shown that obesity constitutes a risk factor for *H. pylori* infection (44, 45, 62). A recent cohort study showed that *H. pylori* infection increased the risk of gastric cancer in participants with BMI <25 kg/m^2^, whereas infection was not associated with cancer risk in participants with BMI ≥ 25 kg/m^2^ (63) and it could be speculated that the seemingly protective effect of overweight on gastric cancer risk could be mediated through less severe *H. pylori* infection. However, the association between *H. pylori*, body weight and underlying mechanisms warrants further research before conclusions can be made regarding a link between *H. pylori*, body weight and disease development.

### Effects on Cardiometabolic Risk Factors

A significant reduction in LDL-C was found after 12 weeks of FRB compared with RW and the effect size (6%) was in the similar range to what has been achieved in studies investigating the effect of oat β-glucan (64). At the follow up examination in week 24, the difference in LDL-C observed after 12 weeks had disappeared, which strongly support the cholesterol lowering was a result of the FRB treatment. Wholegrain rye has previously been shown to reduce LDL-C (38, 65, 66), however few studies have investigated the effect of wholegrain rye specifically, but rather investigated mixtures of wholegrains or wholegrain wheat, hence more studies investigating the effect of rye is needed to confirm the effect (67). The cholesterol lowering effect of oats has been attributed to the high content of viscous fibers, and it may be that the cholesterol lowering effect of rye is caused by soluble arabinoxylans (37, 64, 68). It has been shown that about 80% of the variation in viscosity of water soluble dietary fiber in wholegrain rye flour is due to water soluble arabinoxylan (69). In contrast to wheat, the water soluble arabinoxylans in rye are more resistant to xylanase activity, and therefore the viscosity is retained to a larger extent throughout different food processes (70). Intake of rye, compared to wheat, has been shown to cause significantly higher viscosity of ileal digesta in pigs (71). Based on this, we hypothesize that wholegrain rye may lower LDL-C due to increased viscosity formed by its water soluble arabinoxylans, in analogy to what has been found for beta-glucans from oats and barley (72).

Wholegrain consumption has been linked to reductions in low-grade systemic inflammation, but the evidence from interventions is limited and has produced conflicting results and few studies have been conducted solely on rye (73–76). One of the mechanisms suggested to be involved in the link between the intake of wholegrain or other fiber- rich foods and inflammation is reductions in gut permeability (77). However, we found no effect of the intervention diet on zonulin, a biomarker of gut permeability. Nevertheless, this is in line with results from a recent study that also found reduced low grade inflammation but no effects on gut permeability following a intervention comparing mixed wholegrain with mixed refined grain (30), suggesting that other mechanisms could be involved. It should be noted that the difference between interventions found in the present study appears to be driven mainly by an increase in hs-CRP concentration in the RW groups, indicating an adverse effect of the RW diet rather than a positive effect of the FRB diet. Consumption of refined cereals has been associated with higher concentration of inflammatory markers in observational studies (78), and it is possible that the introduction of a relatively large amount of refined wheat in a population not accustomed to consumption of refined wheat has caused the increase in hs-CRP.

The increase in systemic zonulin seen in both of the intervention groups might be an effect the protein present in both of the diets. It has previously been shown that intestinal exposure to gliadin upregulates zonulin expression (79).

Previous intervention studies with high-fiber rye has mainly been conducted in northern Europe, where rye is habitually consumed (67). The present study is, to our knowledge, the first study to investigate metabolic effects of high-fiber rye food consumption in a population with little or no rye in the habitual diet. While this has provided the opportunity to test the effect of rye without the influence of a background diet rich in rye, this is likely the reason for the apparently lower compliance than what could have been expected in a population more familiar with the taste of rye, by assessment of plasma AR concentration profiles after intervention (39, 80). The daily amount of intervention products was high, especially considering that the study population were not accustomed to consuming this type of products. However, results from a pilot study indicated that high amounts of intervention foods were required to reach the desired effect, and the participants in the pilot study showed markedly better compliance than the participants of the present study. Nevertheless, the clear trend of a group difference in risk markers during the intervention and absence of this difference at the follow in week 24 clearly supports a causal link between the intervention and the reduction in risk markers, despite a lower than expected compliance. It cannot be ruled out that the use of complete cases analysis, rather than intention to treat analysis, may have overestimated the effect of the intervention and we cannot rule out that some bias was introduced due to this approach. However, as complete cases analysis was the defined analysis strategy for the study based on the design and dimensioning of the study.

## Conclusion

In conclusion, the results of the study did not support any effect of the fermented rye bran vs. refined wheat on *H. pylori* infection. However, we cannot rule out whether this is related to the methodology used to assess the infection rate or the low compliance. Baseline data showed lower breath test values among overweight subjects indicating lower *H. pylori* bacterial loads, which is in line with a recent cross-sectional study and warrants further research. The FRB diet lowered LDL-C, while the RW diet increased hs-CRP, which adds to the growing evidence of a positive effect of rye consumption vs. refined wheat on important risk markers of CVDs and provides an interesting tool for prevention.

## Data Availability Statement

The datasets presented in this article are not readily available because the datasets contains person sensitive information, but the datasets are available from the corresponding author on reasonable request. Requests to access the datasets should be directed to Kia Nøhr Iversen, kia.nohr@chalmers.se.

## Ethics Statement

The studies involving human participants were reviewed and approved by Institutional Review Board at Fudan University. The patients/participants provided their written informed consent to participate in this study.

## Author Contributions

AJ, GHa, PÅ, RL, and TJ designed the study. KX, YL, ZQ, and CD conducted the study. KX and GHe provided essential materials. KI performed statistical analysis. KI and RL wrote the paper. MM contributed to drafting of the introduction. RL had responsibility for the final content. All authors contributed to the article and approved the submitted version.

## Conflict of Interest

RL is the scientific leader of Nordic Rye Forum, which is a collaboration platform between academia, institutes and food industry. The platform is funded by industry membership fees. RL has received project grants from industry to conduct specific research, unrelated to the present study. GHa has been working as an adviser for Barilla. GHa, PÅ and AJ have a patent associated with a possible impact of fermented rye on *H. pylori* binding to gastric mucosa. The remaining authors declare that the research was conducted in the absence of any commercial or financial relationships that could be construed as a potential conflict of interest.
