# Supplementary material for: Impact of a Fermented High-Fiber Rye Diet on Helicobacter pylori and Cardio-Metabolic Risk Factors: A Randomized Controlled Trial Among Helicobacter pylori-Positive Chinese Adults
Source: Front Nutr. 2021 Jan 15;7:608623. doi: 10.3389/fnut.2020.608623 (PMC7844128; doi:10.3389/fnut.2020.608623)
Supplement: Supplementary file 1 [file Data_Sheet_1.docx]

Supplementary Material

**Supplementary Table 1:** Antibodies towards virulence factors of *Helicobacter pylori* measured in all participants who completed the 12-week intervention. Data is the absolute number of positive participants and the percentage of positive participants.

|  |  |  | Anti-CagA | | | Anti-UreA | | | Anti-UreB | | | Anti-VacA | | |
| --- | --- | --- | --- | --- | --- | --- | --- | --- | --- | --- | --- | --- | --- | --- |
|  |  |  | Week 0 | Week 6 | week12 | Week 0 | Week 6 | Week 12 | Week 0 | Week 6 | Week 12 | Week 0 | Week 6 | Week 12 |
| Completed participants | All (n=79/78)^1^ | FRB | 57  (72 %) | 59  (75 %) | 51  (65 %) | 68  (86 %) | 67  (85 %) | 57*  (73 %) | 72  (91 %) | 69  (87 %) | 69  (88 %) | 52  (66 %) | 50  (63 %) | 43  (55 %) |
|  |  | RW | 47  (60 %) | 43  (55 %) | 53  (71 %) | 53  (68 %) | 58  (74 %) | 54  (72 %) | 66  (85 %) | 64  (82 %) | 59  (79 %) | 39  (50 %) | 38  (49 %) | 45  (60 %) |
|  | Over-weight^2^ (n=40/34)^1^ | FRB | 27  (68 %) | 30  (75 %) | 26  (65 %) | 36  (90 %) | 34  (85 %) | 31  (78 %) | 38  (95 %) | 36  (90 %) | 37  (93 %) | 26  (65 %) | 24  (60 %) | 20  (50 %) |
|  |  | RW | 21  (62 %) | 20  (59 %) | 19  (59 %) | 22  (65 %) | 27  (79 %) | 22  (69 %) | 31  (91 %) | 29  (85 %) | 24  (75 %) | 16  (47 %) | 16  (47 %) | 17  (53 %) |
|  | Normal weight^3^ (n=44/39)^1^ | FRB | 30  (77 %) | 29  (74 %) | 25  (66 %) | 32  (82 %) | 33  (85 %) | 26  (68 %) | 34  (87 %) | 33  (85 %) | 32  (84 %) | 26  (67 %) | 26  (67 %) | 23  (61 %) |
|  |  | RW | 26  (59 %) | 23  (52 %) | 34*  (79 %) | 31  (70 %) | 31  (70 %) | 32  (74 %) | 35  (80 %) | 35  (80 %) | 35  (81 %) | 23  (52 %) | 22  (50 %) | 28  (65 %) |

*different from baseline, p<0.05, in McNemar’s test. ^1^n=FRB/RW, ^2^BMI>24 kg/m^2^, ^3^ BMI≤24 kg/m^2^. FRB, fermented rye bran; RW, refined wheat; anti-CagA, antibody for cytotoxin-associated gene A; anti-VacA, antibody for vacuolating cytotoxin A; anti-UreA, antibody for urease A; anti-UreB, antibody for urease B.

**Supplementary Table 2:** Antibodies towards virulence factors of *Helicobacter pylori* measured in participants who completed the 12-week intervention and attended the follow-up visit at week 24. Data is the absolute number of positive participants and the percentage of positive participants

|  |  |  | Anti-CagA | | | | Anti-UreA | | | | Anti-UreB | | | | Anti-VacA | | | |
| --- | --- | --- | --- | --- | --- | --- | --- | --- | --- | --- | --- | --- | --- | --- | --- | --- | --- | --- |
|  |  |  | Week 0 | Week 6 | Week 12 | Week 24 | Week 0 | Week 6 | Week 12 | Week 24 | Week 0 | Week 6 | Week 12 | Week 24 | Week 0 | Week 6 | Week 12 | Week 24 |
| Completed participants | All (n=60/57)^1^ | FRB | 43  (72 %) | 45  (75 %) | 38  (64 %) | 36  (60 %) | 52  (87 %) | 51  (85 %) | 43  (73 %) | 39*  (65 %) | 55  (92 %) | 52  (87 %) | 53  (90 %) | 52  (87 %) | 40  (67 %) | 40  (67 %) | 33  (56 %) | 32  (53 %) |
|  |  | RW | 37  (65 %) | 33  (58 %) | 42  (75 %) | 26*  (46 %) | 40  (70 %) | 45  (79 %) | 43  (77 %) | 30*  (53 %) | 52  (91 %) | 49  (86 %) | 47  (84 %) | 43*  (75 %) | 31  (54 %) | 30  (53 %) | 37  (66 %) | 22*  (39 %) |
|  | Over-weight^2^ (n=31/30)^1^ | FRB | 20  (65 %) | 23  (74 %) | 20  (65 %) | 19  (61 %) | 28  (90 %) | 27  (87 %) | 24  (77 %) | 22*  (71 %) | 30  (97 %) | 28  (90 %) | 29  (94 %) | 27  (87 %) | 19  (61 %) | 19  (61 %) | 16  (52 %) | 17  (55 %) |
|  |  | RW | 18  (60 %) | 16  (53 %) | 17  (59 %) | 12  (40 %) | 19  (63 %) | 23  (77 %) | 20  (69 %) | 13  (43 %) | 28  (93 %) | 25  (83 %) | 22  (76 %) | 21*  (70 %) | 13  (43 %) | 13  (43 %) | 16  (55 %) | 8  (27 %) |
|  | Normal weight^3^ (n=29/27)^1^ | FRB | 23  (79 %) | 22  (76 %) | 18  (64 %) | 17  (59 %) | 24  (83 %) | 24  (83 %) | 19  (68 %) | 17  (59 %) | 25  (86 %) | 24  (83 %) | 23  (82 %) | 25  (86 %) | 21  (72 %) | 21  (72 %) | 17  (61 %) | 16  (55 %) |
|  |  | RW | 19  (70 %) | 17  (63 %) | 25*  (93 %) | 14  (52 %) | 21  (78 %) | 22  (81 %) | 23  (85 %) | 17  (63 %) | 24  (89 %) | 24  (89 %) | 25  (93 %) | 22  (81 %) | 18  (67 %) | 17  (63 %) | 22  (81 %) | 14  (52 %) |

* different from baseline, p<0.05, in McNemar’s test. ^1^n=FRB/RW, ^2^BMI>24 kg/m^2^, ^3^ BMI≤24 kg/m^2^. FRB, fermented rye bran; RW, refined wheat; anti-CagA, antibody for cytotoxin-associated gene A; anti-VacA, antibody for vacuolating cytotoxin A; anti-UreA, antibody for urease A; anti-UreB, antibody for urease B.

**Supplementary Table 3:** Effects on clinical outcomes at baseline, after 6 and 12 weeks of dietary intervention with fermented rye bran (FRB) or refined wheat (RW) products among all overweight participants (BMI>24 kg/m^2^) who completed the 12-week intervention. n: FRB=41, RW=34 unless otherwise stated^1^. Data is mean±SD (median).

|  | | **Week 0** | **Week 6** | **Week 12** | **∆ between groups. week 6** | | **∆ between groups. week 12** | |
| --- | --- | --- | --- | --- | --- | --- | --- | --- |
|  |  |  |  |  | Model 1 | Model 2 | Model 1 | Model2 |
| *Body weight (kg)* | | | | | | | | |
|  | FRB | 73.8±12.2  (73.0) | 73.3±12.4  (72.6) | 73.4±12.6  (73.0) | 0.377 | 0.421 | 0.785 | 0.832 |
|  | RW | 74.0±14.4  (71.0) | 74.1±15.0  (69.3) | 73.3±14.8  (68.9) |  |  |  |  |
| *Body fat (%)* | | | | | | | | |
|  | FRB | 32.5±6.1 | 32.3±6.7 | 31.7±5.5 | 0.796 | 0.748 | 0.232 | 0.266 |
|  | RW | 35.1±3.9 | 34.7±4.14 | 34.5±4.2 |  |  |  |  |
| *Total cholesterol (mmol/L)* | | | | | | | | |
|  | FRB | 5.08±0.90 | 4.89±0.83 | 4.88±0.95 | 0.486 | 0.495 | 0.375 | 0.647 |
|  | RW | 5.21±0.89 | 5.05±0.71 | 5.14±0.67 |  |  |  |  |
| *HDL cholesterol (mmol/L)* | | | | | | | | |
|  | FRB | 1.47±0.37  (1.45) | 1.36±0.32  (1.35) | 1.39±0.30  (1.35) | 0.165 | 0.201 | 0.605 | 0.313 |
|  | RW | 1.37±0.36  (1.36) | 1.22±0.29  (1.19) | 1.29±0.31  (1.24) |  |  |  |  |
| *LDL cholesterol (mmol/L)* | | | | | | | | |
|  | FRB | 3.08±0.64 | 2.81±0.58 | 2.72±0.69 | 0.867 | 0.735 | 0.194 | 0.307 |
|  | RW | 3.16±0.59 | 2.87±0.48 | 2.94±0.43 |  |  |  |  |
| *Triglycerides (mmol/L)* | | | | | | | | |
|  | FRB | 1.28±0.65  (1.17) | 1.39±0.81  (1.16) | 1.58±1.32  (1.28) | 0.063 | 0.093 | 0.841 | 0.843 |
|  | RW | 1.48±0.65  (1.41) | 1.74±0.78  (1.67) | 1.76±1.10  (1.38) |  |  |  |  |
| *Glucose (mmol/L)* | | | | | | | | |
|  | FRB | 5.71±0.92 | 5.71±0.64 | 5.56±0.86 | 0.883 | 0.939 | 0.813 | 0.815 |
|  | RW | 6.19±1.11 | 5.91±1.12 | 5.88±1.18 |  |  |  |  |
| *Insulin (pmol/L)* | | | | | | | | |
|  | FRB | 85.8±44.3  (72.6) | 88.6±58.2  (72.5) | 95.3±59.0  (80.8) | 0.744 | 0.916 | 0.838 | 0.987 |
|  | RW | 93.2±43.8  (85.4) | 103.4±67.4  (87.7) | 94.9±45.4  (90.1) |  |  |  |  |
| *C-peptide (nmol/L)* | | | | | | | | |
|  | FRB | 0.80±0.29  (0.76) | 0.82±0.29  (0.74) | 0.94±0.37  (0.84) | 0.780 | 0.800 | 0.510 | 0.663 |
|  | RW | 0.93±0.29  (0.88) | 0.95±0.35  (0.85) | 0.96±0.26  (0.93) |  |  |  |  |
| *HOMA-IR* | | | | | | | | |
|  | FRB | 3.7±2.1  (3.2) | 3.7±2.4  (2.9) | 4.0±2.7  (3.2) | 0.869 | 0.967 | 0.879 | 0.957 |
|  | RW | 4.3±2.2  (3.9) | 4.7±3.7  (4.1) | 4.3±2.8  (4.1) |  |  |  |  |
| *HbA1C (%)* | | | | | | | | |
|  | FRB | 5.55±0.38 | 5.86±0.43 | 5.84±0.40 | 0.230 | 0.299 | 0.499 | 0.445 |
|  | RW | 5.83±0.74 | 6.09±0.64 | 6.08±0.65 |  |  |  |  |
| *Apolipoprotein A (g/L)* | | | | | | | | |
|  | FRB | 1.34±0.23  (1.31) | 1.34±0.21  (1.31) | 1.32±0.18  (1.31) | 0.930 | 0.563 | 0.630 | 0.658 |
|  | RW | 1.28±0.22  (1.29) | 1.28±0.21  (1.27) | 1.30±0.22  (1.28) |  |  |  |  |
| *Apolipoprotein B (g/L)* | | | | | | | | |
|  | FRB | 1.13±0.25 | 1.19±0.27 | 1.18±0.24 | 0.916 | 0.856 | 0.437 | 0.537 |
|  | RW | 1.20±0.22 | 1.23±0.18 | 1.25±0.22 |  |  |  |  |
| *Apolipoprotein A /Apolipoprotein B* | | | | | | | | |
|  | FRB | 1.23±0.28 | 1.18±0.36 | 1.18±0.31 | 0.815 | 0.493 | 0.808 | 0.549 |
|  | RW | 1.10±0.24 | 1.07±0.25 | 1.07±0.31 |  |  |  |  |
| *Apolipoprotein E (g/L)* | | | | | | | | |
|  | FRB | 32.53±5.06 | 32.96±4.59 | 33.11±5.33 | 0.872 | 0.946 | 0.853 | 0.970 |
|  | RW | 34.63±4.23 | 34.37±4.43 | 34.91±5.22 |  |  |  |  |
| *Lipoprotein (a) (mg/L)* | | | | | | | | |
|  | FRB | 191.1±171.2  (133.5) | 216.7±210.5  (131.0) | 222.5±237.4  (126.0) | 0.548 | 0.502 | 0.713 | 0.493 |
|  | RW | 202.0±190.7  (120.0) | 219.8±270.6  (105.0) | 242.6±272.5  (112.0) |  |  |  |  |
| *hs-CRP (mg/dl) (values ≥1 mg/dl removed)* | | | | | | | | |
|  | FRB | 0.170±0.204  (0.095) | 0.124±0.156  (0.060) | 0.173±0.215  (0.080) | 0.001 | 0.002 | 0.006 | 0.006 |
|  | RW | 0.230±0.180  (0.163) | 0.238±0.156  (0.249) | 0.279±0.222  (0.205) |  |  |  |  |
| *Zonulin (pg/ml)* | | | | | | | | |
|  | FRB | 282.45±88.13  (255.84) | 367.01±100.91  (366.13) | 347.56±87.82  (341.76) | 0.908 | 0.918 | 0.989 | 0.999 |
|  | RW | 284.73±83.43  (257.80) | 365.89±103.39  (356.82) | 350.14±99.74  (356.93) |  |  |  |  |

^1^Number of participants, when deviating from 41/34 (FRB/RW). Body weight: wk6=41/31, C-peptide: wk12=41/33, lipoprotein (a): wk6=40/31, hs-CRP: wk0=39/32, wk6=39/32, wk12=40/34, zonulin: wk6=40/32, wk12=40/34.

HDL, high density lipoprotein; LDL, low density lipoprotein; HOMA-IR, homeostasis model assessment insulin resistance; HbA1C, hemoglobin A1c; hs-CRP, high sensitivity C-reactive protein;

**Supplementary Table 4:** Effects on clinical outcomes at baseline, after 6 and 12 weeks of dietary intervention with fermented rye bran (FRB) or refined wheat (RW) products among all normal weight participants (BMI≤24 kg/m^2^) who completed the 12-week intervention. n: FRB=39, RW=44 unless otherwise stated^1^. Data is mean±SD (median).

|  | | Week 0 | Week 6 | Week 12 | ∆ between groups. week 6 | | ∆ between groups. week 12 | |
| --- | --- | --- | --- | --- | --- | --- | --- | --- |
|  |  |  |  |  | Model 1 | Model 2 | Model 1 | Model2 |
| *Body weight (kg)* | | | | | | | | |
|  | FRB | 55.2±5.6 | 55.0±5.8 | 54.7±5.9 | 0.805 | 0.762 | 0.547 | 0.638 |
|  | RW | 54.5±6.2 | 54.2±6.5 | 54.3±6.3 |  |  |  |  |
| *Body fat (%)* | | | | | | | | |
|  | FRB | 29.0±5.2 | 28.2±5.0 | 28.2±4.8 | 0.606 | 0.756 | 0.300 | 0.371 |
|  | RW | 28.7±4.2 | 27.9±4.2 | 27.4±4.3 |  |  |  |  |
| *Total cholesterol (mmol/L)* | | | | | | | | |
|  | FRB | 5.24±1.17  (5.18) | 4.92±1.01  (4.81) | 5.09±1.21  (4.86) | 0.124 | 0.083 | 0.066 | 0.045 |
|  | RW | 5.09±1.08  (4.94) | 5.01±1.09  (4.83) | 5.24±1.17  (5.32) |  |  |  |  |
| *HDL cholesterol (mmol/L)* | | | | | | | | |
|  | FRB | 1.71±0.36 | 1.55±0.33 | 1.53±0.34 | 0.837 | 0.791 | 0.468 | 0.269 |
|  | RW | 1.70±0.38 | 1.52±0.34 | 1.55±0.32 |  |  |  |  |
| *LDL cholesterol (mmol/L)* | | | | | | | | |
|  | FRB | 3.14±0.90 | 2.75±0.73 | 2.85±0.85 | 0.029 | 0.016 | 0.019 | 0.015 |
|  | RW | 3.04±0.83 | 2.90±0.80 | 3.06±0.87 |  |  |  |  |
| *Triglycerides (mmol/L)* | | | | | | | | |
|  | FRB | 1.00±0.38  (0.86) | 1.10±0.51  (1.01) | 1.40±0.76  (1.22) | 0.288 | 0.360 | 0.035 | 0.032 |
|  | RW | 0.99±0.48  (0.85) | 1.03±0.48  (0.93) | 1.18±0.48  (1.05) |  |  |  |  |
| *Glucose (mmol/L)* | | | | | | | | |
|  | FRB | 5.29±0.74 | 5.35±0.42 | 5.23±0.59 | 0.483 | 0.498 | 0.389 | 0.146 |
|  | RW | 5.40±0.70 | 5.44±0.46 | 5.36±0.52 |  |  |  |  |
| *Insulin (pmol/L)* | | | | | | | | |
|  | FRB | 53.7±21.1  (52.1) | 57.0±21.8  (52.6) | 66.7±48.5  (48.0) | 0.080 | 0.051 | 0.756 | 0.828 |
|  | RW | 55.9±27.5  (51.2) | 53.8±30.9  (48.0) | 61.2±29.3  (55.8) |  |  |  |  |
| *C-peptide (nmol/L)* | | | | | | | | |
|  | FRB | 0.59±0.16  (0.56) | 0.63±0.18  (0.55) | 0.75±0.37  (0.62) | 0.348 | 0.292 | 0.628 | 0.804 |
|  | RW | 0.59±0.16  (0.56) | 0.61±0.23  (0.57) | 0.70±0.25  (0.65) |  |  |  |  |
| *HOMA-IR* | | | | | | | | |
|  | FRB | 2.2±1.1  (2.1) | 2.3±0.9  (2.2) | 2.68±2.27  (1.92) | 0.143 | 0.095 | 0.951 | 0.902 |
|  | RW | 2.3±1.2  (1.9) | 2.2±1.4  (2.1) | 2.43±1.18  (2.19) |  |  |  |  |
| *HbA1C %* | | | | | | | | |
|  | FRB | 5.45±0.39 | 5.68±0.32 | 5.71±0.31 | 0.269 | 0.269 | 0.278 | 0.176 |
|  | RW | 5.37±0.32 | 5.67±0.35 | 5.71±0.34 |  |  |  |  |
| *Apolipoprotein A (g/L)* | | | | | | | | |
|  | FRB | 1.42±0.23 | 1.39±0.23 | 1.38±0.22 | 0.637 | 0.701 | 0.494 | 0.395 |
|  | RW | 1.43±0.21 | 1.38±0.23 | 1.41±0.23 |  |  |  |  |
| *Apolipoprotein B (g/L)* | | | | | | | | |
|  | FRB | 1.12±0.30  (1.03) | 1.14±0.29  (1.13) | 1.13±0.31  (1.13) | 0.204 | 0.192 | 0.075 | 0.090 |
|  | RW | 1.08±0.25  (1.04) | 1.17±0.21  (1.14) | 1.20±0.29  (1.19) |  |  |  |  |
| *Apolipoprotein A /Apolipoprotein B* | | | | | | | | |
|  | FRB | 1.36±0.42 | 1.33±0.50 | 1.31±0.41 | 0.147 | 0.163 | 0.205 | 0.272 |
|  | RW | 1.38±0.31 | 1.21±0.27 | 1.25±0.38 |  |  |  |  |
| *Apolipoprotein E (g/L)* | | | | | | | | |
|  | FRB | 33.65±6.40 | 33.42±6.68 | 34.01±6.09 | 0.493 | 0.451 | 0.213 | 0.122 |
|  | RW | 33.12±6.16 | 32.21±6.01 | 32.67±5.15 |  |  |  |  |
| *Lipoprotein (a) (mg/L)* | | | | | | | | |
|  | FRB | 237.3±192.4  (155.0) | 266.8±237.4  (193.0) | 299.1±304.0  (163.0) | 0.626 | 0.805 | 0.697 | 0.578 |
|  | RW | 163.3±155.7  (124.0) | 199.5±250.3  (127.0) | 188.0±202.1  (122.0) |  |  |  |  |
| *hs-CRP (mg/dl) (values ≥1 mg/dl removed)* | | | | | | | | |
|  | FRB | 0.093±0.154  (0.047) | 0.062±0.070  (0.032) | 0.072±0.088  (0.040) | 0.825 | 0.786 | 0.170 | 0.204 |
|  | RW | 0.059±0.083  (0.031) | 0.054±0.056  (0.028) | 0.078±0.077  (0.048) |  |  |  |  |
| *Zonulin (pg/ml)* | | | | | | | | |
|  | FRB | 263.91±88.76  (237.91) | 348.72±80.72  (348.29) | 381.33±98.46  (366.89) | 0.763 | 0.788 | 0.600 | 0.744 |
|  | RW | 288.51±107.25  (310.85) | 349.14±96.21  (318.53) | 367.84±103.5  (364.03) |  |  |  |  |

^1^Number of participants, when deviating from 39/44 (FRB/RW). Body weight: wk12=38/44, body fat %: wk0=38/43, wk12=38/44, insulin: wk12=38/43, C-peptide: wk12=38/43, HOMA-IR: wk12=38/43, lipoprotein (a): wk6=37/43, hs-CRP: wk0=38/44, wk6=38/42, wk12=38/44, zonulin: wk6=38/43.

HDL, high density lipoprotein; LDL, low density lipoprotein; HOMA-IR, homeostasis model assessment insulin resistance; HbA1C, hemoglobin A1c; hs-CRP, high sensitivity C-reactive protein;

**Supplementary Table 5:** Effects on clinical outcomes at baseline and after 12 weeks of dietary intervention with fermented rye bran (FRB) or refined wheat (RW) products, as well as 12 weeks post intervention (week 24) among all participants who completed the 12-week intervention and attended the follow up visit at week 24. n(FRB/RW): wk0 and week 12=51/51, wk24=50/51 unless otherwise stated^1^. Data is mean±SD (median).

|  | | Week 0 | Week 12 | Week 24 | ∆ between groups. week 12 | | ∆ between groups. week 24 | |
| --- | --- | --- | --- | --- | --- | --- | --- | --- |
|  |  |  |  |  | Model 1 | Model 2 | Model 1 | Model 2 |
| *Body weight (kg)* | | | | | | | | |
|  | FRB | 63.5±14.1  (60.0) | 63.4±14.7  (58.8) | 63.0±13.9  (58.6) | 0.560 | 0.730 | 0.333 | 0.516 |
|  | RW | 65.1±14.5  (62.6) | 64.5±14.6  (61.2) | 64.3±15.3  (60.8) |  |  |  |  |
| *Body fat (%)* | | | | | | | | |
|  | FRB | 30.5±5.7 | 29.9±5.4 | 30.4±5.9 | 0.842 | 0.789 | 0.785 | 0.888 |
|  | RW | 32.2±5.2 | 31.4±5.2 | 32.0±5.1 |  |  |  |  |
| *Bacterial load (DOB ‰)* | | | | | | | | |
|  | FRB | 18.6±14.0  (14.8) | 21.2±20.1  (15.2) | 16.5±15.9  (12.2) | 0.083 | 0.096 | 0.334 | 0.342 |
|  | RW | 17.5±12.3  (13.2) | 27.4±23.3  (21.9) | 15.9±12.0  (16.1) |  |  |  |  |
| *Triglycerides (mmol/L)* | | | | | | | | |
|  | FRB | 1.21±0.64  (1.06) | 1.60±1.21  (1.28) | 1.41±1.39  (1.13) | 0.536 | 0.338 | 0.074 | 0.124 |
|  | RW | 1.31±0.63  (1.07) | 1.59±0.96  (1.31) | 1.18±0.52  (1.12) |  |  |  |  |
| *Glucose (mmol/L)* | | | | | | | | |
|  | FRB | 5.51±0.92 | 5.46±0.84 | 5.44±0.74 | 0.511 | 0.470 | 0.832 | 0.677 |
|  | RW | 5.92±1.09 | 5.79±0.97 | 5.68±1.13 |  |  |  |  |
| *Insulin (pmol/L)* | | | | | | | | |
|  | FRB | 75.0±41.8  (61.3) | 79.7±48.1  (65.1) | 70.9±85.8  (55.9) | 0.997 | 0.585 | 0.459 | 0.303 |
|  | RW | 74.3±38.7  (66.0) | 75.5±38.0  (69.2) | 64.7±49.5  (52.8) |  |  |  |  |
| *C-peptide (nmol/L)* | | | | | | | | |
|  | FRB | 0.73±0.29  (0.66) | 0.84±0.34  (0.74) | 1.09±0.68  (0.89) | 0.535 | 0.938 | 0.334 | 0.460 |
|  | RW | 0.75±0.26  (0.71) | 0.82±0.27  (0.78) | 1.02±0.40  (0.96) |  |  |  |  |
| *HOMA-IR* | | | | | | | | |
|  | FRB | 3.2±2.1  (2.4) | 3.3±2.3  (2.5) | 3.0±4.1  (2.1) | 0.795 | 0.449 | 0.573 | 0.395 |
|  | RW | 3.3±2.0  (2.8) | 3.4±2.4  (2.9) | 2.9±3.0  (2.1) |  |  |  |  |
| *HbA1C (%)* | | | | | | | | |
|  | FRB | 5.50±0.42  (5.4) | 5.76±0.38  (5.70) | 5.64±0.54  (5.60) | 0.242 | 0.230 | 0.219 | 0.215 |
|  | RW | 5.67±0.64  (5.50) | 5.95±0.57  (5.80) | 5.90±0.81  (5.70) |  |  |  |  |
| *Apolipoprotein A (g/L)* | | | | | | | | |
|  | FRB | 1.38±0.23 | 1.37±0.22 | 1.45±0.29 | 0.493 | 0.733 | 0.770 | 0.479 |
|  | RW | 1.40±0.23 | 1.40±0.25 | 1.44±0.37 |  |  |  |  |
| *Apolipoprotein B (g/L)* | | | | | | | | |
|  | FRB | 1.11±0.26 | 1.17±0.26 | 0.99±0.23 | 0.633 | 0.828 | 0.498 | 0.237 |
|  | RW | 1.14±0.22 | 1.21±0.23 | 0.98±0.19 |  |  |  |  |
| *Apolipoprotein A /Apolipoprotein B* | | | | | | | | |
|  | FRB | 1.31±0.35 | 1.22±0.31 | 1.55±0.38 | 0.912 | 0.750 | 0.752 | 0.816 |
|  | RW | 1.26±0.29 | 1.22±0.38 | 1.49±0.44 |  |  |  |  |
| *Apolipoprotein E (g/L)* | | | | | | | | |
|  | FRB | 33.75±5.93 | 34.15±5.92 | 35.08±6.55 | 0.673 | 0.455 | 0.051 | 0.064 |
|  | RW | 33.94±5.20 | 33.97±5.31 | 33.35±4.57 |  |  |  |  |
| *Lipoprotein (a) (mg/L)* | | | | | | | | |
|  | FRB | 228.1±183.8  (162.0) | 253.6±237.2  (156.0) | 278.4±277.8  (158.0) | 0.384 | 0.193 | 0.979 | 0.460 |
|  | RW | 191.4±198.4  (105.0) | 222.5±269.9  (109.0) | 238.9±271.0  (150.0) |  |  |  |  |
| *Zonulin (pg/ml)* | | | | | | | | |
|  | FRB | 277.40±90.03  (256.31) | 364.65±90.03  (354.72) | 410.65±107.72  (406.04) | 0.773 | 0.805 | 0.032 | 0.034 |
|  | RW | 289.22±97.30  (281.26) | 359.89±97.48  (350.79) | 454.54±93.15  (461.04) |  |  |  |  |

^1^Deviating sample sizes (FRB/RW): Insulin: wk12=20/38, C-peptide: wk12= 20/38, HOMA-IR: wk12=20/38, Lipoprotein (a): wk6=19/38, hs-CRP: wk6=21/37.

HOMA-IR, homeostasis model assessment insulin resistance; HbA1C, hemoglobin A1c;


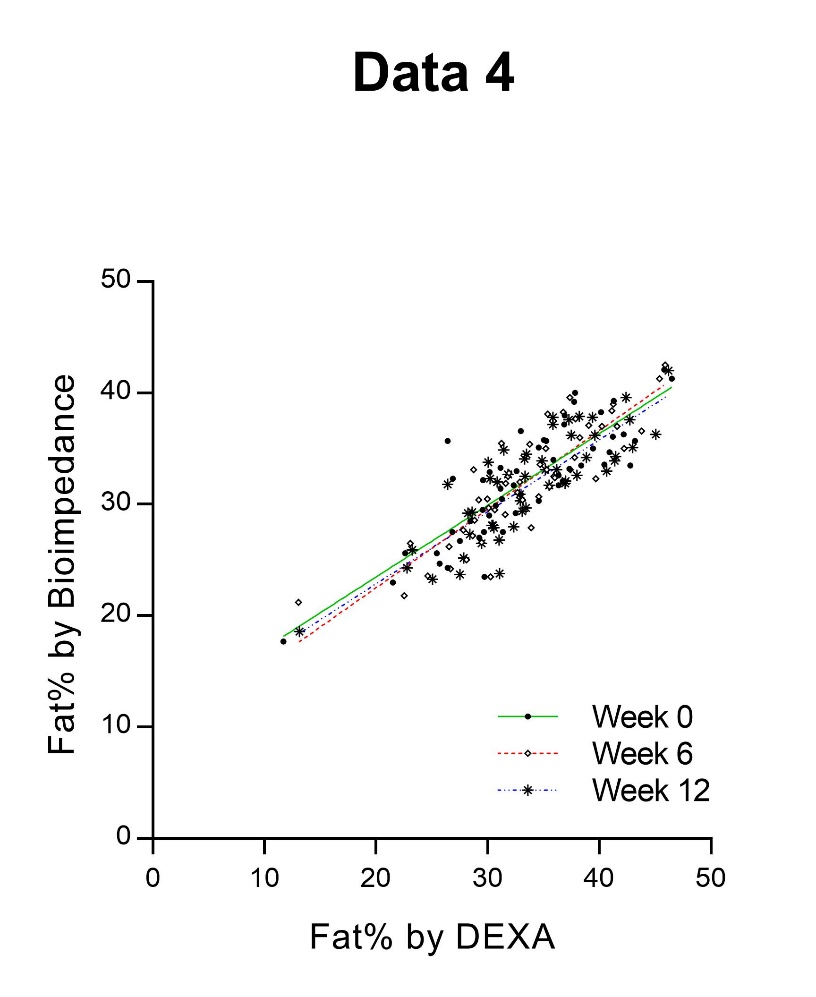


**Supplementary Figure 1:** Correlation between fat % measured by dual energy x-ray absorptiometry (DEXA) and bioimpedance. Pearson correlation coefficient: wk0=0.8397, wk6=0.8699, wk12=0.8370. p<0.001 at all three timepoints.
